# Association of plasma brain-derived neurotrophic factor levels and frailty in community-dwelling older adults

**DOI:** 10.1038/s41598-022-19706-3

**Published:** 2022-11-03

**Authors:** Eun Roh, Soon Young Hwang, Eyun Song, Min Jeong Park, Hye Jin Yoo, Sei Hyun Baik, Miji Kim, Chang Won Won, Kyung Mook Choi

**Affiliations:** 1 Division of Endocrinology and Metabolism, Department of Internal Medicine, Hallym University Sacred Heart Hospital, Anyang, 14068 Republic of Korea; 2 Department of Biostatistics, Korea University College of Medicine, Seoul, 08308 Republic of Korea; 3 Division of Endocrinology and Metabolism, Department of Internal Medicine, Korea University Guro Hospital, Korea University College of Medicine, 148 Gurodong-ro, Guro-gu, Seoul, 08308 Republic of Korea; 4 East-West Medical Research Institute, Kyung Hee University, Seoul, 02447 Republic of Korea; 5 Department of Family Medicine, College of Medicine, Kyung Hee University, Seoul, 02447 Republic of Korea

**Keywords:** Biomarkers, Endocrinology

## Abstract

Brain-derived neurotrophic factor (BDNF), an exercise-induced neurotrophin, is an important factor in memory consolidation and cognitive function. This study evaluates the association between plasma BDNF levels and frailty in community-dwelling older adults. Plasma BDNF levels were analyzed in a total of 302 individuals aged 70–84 years from the Korean Frailty and Aging Cohort Study. There were 30 (9.9%) participants with frailty. They were older and had a higher prevalence of dementia and depression than those without frailty. There were no differences in the proportion of male sex between the frail and non-frail groups. Plasma BDNF levels were significantly lower in participants with frailty than in those without frailty. The presence of frailty was significantly associated with plasma BDNF levels (odds ratio 0.508, 95% confidence interval 0.304–0.849) as well as age, hemoglobin, and the presence of dementia, and depression. After adjustment for confounding factors, the significant association between plasma BDNF and frailty was maintained (0.495, 0.281–0.874). This association remained consistent after exclusion of individuals with dementia, depression, stroke, diabetes, and osteoporosis. Plasma BDNF levels were significantly associated with frailty in community-dwelling older adults. Our study may suggest the possible role of BDNF as a novel biomarker of frailty.

## Introduction

Brain-derived neurotrophic factor (BDNF) is the most abundant neurotrophin that regulates neuronal survival, differentiation, and synaptic plasticity in the brain^1^. The amount of plasma BDNF has been considered to partly reflect its secretion in the brain^2^, although other potential sources contribute to its circulating levels including platelets, vascular endothelial and smooth muscle cells, and activated macrophages and lymphocytes^3,4^. Growing evidence has suggested that blood BDNF levels could be used as a biomarker for diagnosis, prognosis, and treatment monitoring of Alzheimer’s disease^5^. Blood BDNF levels are involved in the recurrent and severity of depression^6^. In addition, it is well established that lower levels of circulating BDNF are related to a high risk of stroke^7^ and poor recovery after stroke^8^. Furthermore, low levels of blood BDNF accompany impaired glucose metabolism^9^ and low bone mineral density^10^.

Regular exercise is known to reduce the risk of cardiometabolic diseases, dementia, and depression^11–13^. Recent studies suggested that BDNF may be a key mediator linking vascular and metabolic benefits of exercise with protection from neurodegenerative diseases^11^. In fact, little has been known about the sources and mechanisms underlying exercise-induced changes in circulation levels of BDNF^14^. BDNF is identified as a contraction-inducible protein in skeletal muscle because it has been shown to be produced by skeletal muscle during exercise and enhance lipid oxidation in skeletal muscle via activation of AMP-activated protein kinase (AMPK)^15^. However, the BDNF synthesized by the skeletal muscle during physical exercise acts on the muscle in an autocrine and paracrine manner^15^. Interestingly, exercise provokes synthesis of fibronectin type III domain-containing protein 5 (FNDC5) and its cleaved, circulating form irisin, which may cross the blood–brain barrier and induces BDNF production^16^. In obese animals, administration of BDNF mimetic induces mitochondrial biogenesis, promotes lipid oxidation in skeletal muscle, and alleviates metabolic derangement^17^. Serum BDNF levels are significantly elevated in humans in response to exercise training^18^. Recent human studies demonstrated the correlation of blood BDNF and skeletal muscle function. Serum BDNF was positively correlated with skeletal muscle strength, but not with muscle mass, in patients with heart failure^19^. Moreover, decline in BDNF was associated with deterioration in muscle strength and physical performance in Japanese hemodialysis patients^20^.

Frailty, a clinical syndrome of decline in reserve and resistance to stressors across multiple physiologic systems, is common in elderly individuals^21^. As such, frailty has been associated with adverse outcomes including prevalent cardiovascular disease (CVD) and mortality^22,23^. Fried et al.^21^ defined a clinical phenotype of frailty, in which three or more of the following five criteria are present: unintentional weight loss, self-reported exhaustion, weakness, slowness, and low physical activity. Therefore, loss of muscle mass and function in elderly individuals can contribute to greater vulnerability to frailty. Despite the possible role of blood BDNF in muscle function, the relationship between circulating BDNF and frailty has not yet been fully elucidated. Exploration off frailty biomarkers may support the early identification of elderly individuals with frailty and easier monitoring during intervention^24^. Therefore, we aimed to investigate whether plasma BDNF levels are correlated with frailty in community-dwelling Korean older adults. We also conducted sensitivity analyses to exclude individuals with BNDF-related comorbidities including dementia, depression, stroke, diabetes, CVD, and osteoporosis.

## Results

### Study subject characteristics

This study included a total of 302 participants with median age 75 years (IQR 73–79 years). Among them, 30 (9.33%) had physical frailty. Baseline characteristics of the study population were shown in Table 1. Frail group had higher age compared to non-frail group. There were no differences in the proportion of male sex, current smoker, regular drinker, and low income; BMI and blood pressure; TC, FPG, HOMA-IR, hs-CRP, hemoglobin, and creatinine; in the prevalence of diabetes, hypertension, dyslipidemia, and obesity between the frail and non-frail groups. However, participants with frailty had higher proportion of dementia (40% vs. 14%, P < 0.001) and depression (43.3% vs. 13.6%, P < 0.001) compared to those without frailty.

**Table 1:** Baseline characteristics of study population according to the presence of frailty.

Variables	Total	No Frailty	Frailty	*P*-value
N	302	272	30	
Age	75 (73, 79)	75 (72, 78)	78.5 (75, 80)	0.001
Male, n (%)	148 (49)	134 (49.3)	14 (46.7)	0.787
BMI, kg/m^2^	25.15 ± 3.37	25.15 ± 3.3	25.13 ± 4.01	0.977
SBP, mmHg	131.76 ± 14.94	132.06 ± 14.79	128.98 ± 16.24	0.284
DBP, mmHg	77.84 ± 9.07	78.05 ± 9.07	75.97 ± 8.95	0.234
Current smoker, n (%)	24 (7.9)	21 (7.7)	3 (10)	0.661
Regular drinking, n (%)	57 (18.9)	54 (19.9)	3 (10)	0.191
Low income, n (%)	110 (36.4)	95 (34.9)	15 (50)	0.104
Total cholesterol	171 (147, 194)	171 (146, 194)	173.5 (153, 194)	0.858
FPG, mg/dL	98 (91, 116)	98 (90.5, 114)	103.5 (93, 123)	0.129
HOMA-IR	1.54 (1.02, 2.66)	1.50 (1.00, 2.63)	1.97 (1.24, 3.07)	0.140
hs-CRP, mg/dL	0.72 (0.46, 1.34)	0.73 (0.47, 1.33)	0.63 (0.42, 2.19)	0.974
Hemoglobin, g/dL	13.63 ± 1.34	13.68 ± 1.28	13.16 ± 1.76	0.124
Creatinine, mg/dL	0.82 (0.7, 0.96)	0.82 (0.7, 0.96)	0.88 (0.72, 1.06)	0.203
BDNF, ng/mL	7.41 (5.03, 11.41)	7.54 (5.19, 11.60)	4.94 (3.02, 7.91)	0.008
Diabetes, n (%)	98 (32.5)	84 (30.9)	14 (46.7)	0.080
Hypertension, n (%)	199 (65.9)	176 (64.7)	23 (76.7)	0.190
Dyslipidemia, n (%)	128 (42.4)	117 (43)	11 (36.7)	0.463
Obesity, n (%)	150 (49.7)	138 (50.7)	12 (40)	0.264
Dementia, n (%)	50 (16.6)	38 (14)	12 (40)	< 0.001
Depression, n (%)	50 (16.6)	37 (13.6)	13 (43.3)	< 0.001

Data are expressed as percentage or as mean (IQR) or mean ± SD, unless otherwise noted. Groups were compared using χ2 test for categorical variables and independent two-sample t-test or Mann-Whiney U-test for continuous variables after log-transformation of skewed variables including plasma BDNF. BDNF, brain-derived neurotrophic factor; BMI, body mass index; DBP, diastolic blood pressure; FPG, fasting plasma glucose; HOMA-IR, homeostasis model assessment-insulin resistance; hs-CRP, high-sensitivity C-reactive protein; SBP, systolic blood pressure.

### Plasma BDNF levels in participants with and without frailty

**Figure 1:**
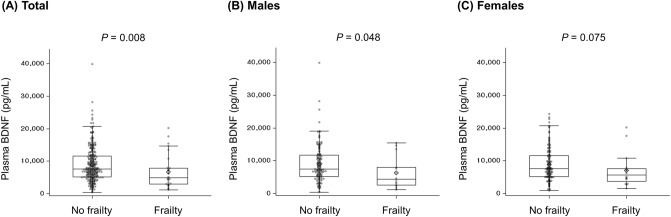
Plasma brain-derived neurotrophic factor levels according to the presence of frailty in total participants (**A**), males (**B**), and females (**C**).

Plasma BDNF levels were significantly lower in participants with frailty than in those without frailty (median [IQR]: 4.94 [3.02, 7.91] vs. 7.54 [5.19, 11.60] ng/mL, P = 0.008) (Table 1, Fig. 1). When divided by sex, plasma BDNF levels were lower in frail subjects compared to control subjects, but not in statistically significant in women (Fig. 1).

### Association between plasma BDNF levels and frailty

Next, we performed univariate and multivariate logistic analyses to identify the association between the presence of frailty and plasma BDNF concentration and metabolic parameters (Table 2). In a univariate analysis, an increase in plasma BDNF was associated with a reduced risk of frailty (odds ratio [OR] 0.508, 95% confidence interval [CI] 0.304–0.849, *P* = 0.01). On the other hand, an increase in age and the presence of dementia or depression were associated with an increased risk of frailty. The association between plasma BDNF and frailty remained in the multivariate analysis after adjustment for confounding factors (OR 0.495, 95% CI 0.281–0.874, *P* = 0.015).

**Table 2:** Association of brain-derived neurotrophic factor or metabolic parameters with the presence of frailty.

	Univariate		Multivariate	
	OR (95% CI)	*P*-value	OR (95% CI)	*P*-value
Age	1.187 (1.067, 1.321)	0.002	1.135 (1.007, 1.278)	0.038
Sex	0.901 (0.423, 1.918)	0.787		
BMI	0.998 (0.893, 1.117)	0.977		
SBP	0.986 (0.961, 1.012)	0.283		
Current smoking	1.328 (0.372, 4.745)	0.662		
Regular drinking	0.449 (0.131, 1.534)	0.201		
Low income	1.863 (0.873, 3.975)	0.108		
TC	1.001 (0.99, 1.012)	0.864		
FPG	1.009 (0.999, 1.02)	0.091		
HOMA-IR	1.015 (0.908, 1.135)	0.789		
hs-CRP	1.066 (0.939, 1.21)	0.322		
Hemoglobin	0.742 (0.556, 0.991)	0.044	0.868 (0.637, 1.182)	0.368
Creatinine	4.45 (0.864, 22.917)	0.074		
BDNF	0.508 (0.304, 0.849)	0.010	0.495 (0.281, 0.874)	0.015
Dementia	4.105 (1.832, 9.199)	0.001	2.253 (0.868, 5.847)	0.095
Depression	4.857 (2.18, 10.82)	< 0.001	3.411 (1.405, 8.281)	0.007

Univariate and multivariate logistic regression analyses for the presence of physical frailty as the dependent variable and plasma BNDF value and metabolic parameters as the independent variables. In a multivariate logistic regression model, all factors that were found to be significant in univariate analyses were used. BDNF, brain-derived neurotrophic factor; BMI, body mass index; CI, confidence interval; FPG, fasting plasma glucose; HOMA-IR, homeostasis model assessment-insulin resistance; hs-CRP, high-sensitivity C-reactive protein; OR, odds ratio; SBP, systolic blood pressure; TC, total cholesterol.

**Figure 2:**
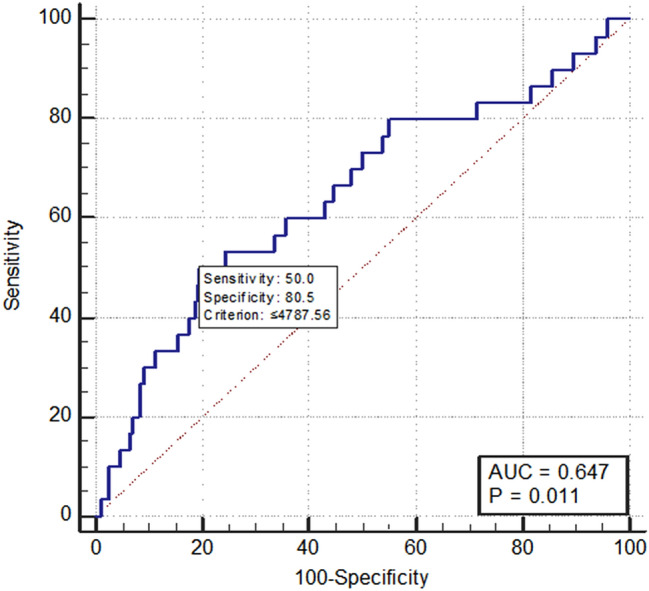
Receiver operating characteristic curves of plasma brain-derived neurotrophic factor levels for estimating frailty.

Altered circulating BDNF levels are known to be related to metabolic and neuropsychiatric disorders including dementia, depression, stroke, diabetes, CVD, and osteoporosis^6–8,10,25^. Therefore, sensitivity analyses were performed by excluding participants with these comorbidities (Supplementary Table 1), and consistent results were obtained.ROC analysis was used to assess the sensitivity and specificity of BDNF in predicting frailty (Fig. 2). The optimal cutoff value of BDNF that predicted frailty was ≤ 4.79 ng/ml, with a sensitivity of 50% and a specificity of 80.5% (the area under the curve 0.647, 95% CI 0.590 to 0.701, *P* = 0.011).

## Discussion

In the present study, plasma BDNF levels were significantly lower in individuals with frailty than in those without among community-dwelling older adults. We also found that plasma BNDF was significantly associated with frailty, regardless of the presence of dementia or depression. Interestingly, the association between plasma BDNF and frailty remained even after exclusion of the effects of dementia, depression, and metabolic disorders, including stroke, diabetes, CVD, and osteoporosis.

BDNF plays a crucial role in the development and functioning of the central nervous system. It is well established that dysfunction of BDNF has been involved in the progression of multiple neurological diseases and psychiatric disorders such as Alzheimer’s disease, stroke, and depression^5–8^. BDNF interacts with receptor tyrosine kinase TrkB and activates downstream intracellular signaling pathways: mainly the phosphatidylinositol 3-kinase (PI3K)/Akt and the mitogen-activated protein kinase/extracellular-signal-regulated kinase (MAPK/ERK) pathways^26^, Furthermore, BDNF is an important modulator of inflammation and has an antioxidant effect with enhancing sestrin2 expression through NF-κB-dependent pathway in rat cortical neurons^27^. Accumulating evidence also suggests that oxidative stress contributes to the dysfunction of BDNF in the pathophysiology of neurodegenerative disorders and psychiatric disorders^28^. Through the regulation of the activity of certain transcription factors, BNDF promotes neuronal differentiation, survival, and regeneration. Thus, potential therapeutic benefits of BDNF has been studied widely in Alzheimer's disease, stroke, and depression^29^. Down-regulation of BDNF results in neuronal susceptibility to oxidative stress and dysfunction induced by neurotoxic amyloid-β in Alzheimer’s disease^11^. Circulating BDNF derives from both central and peripheral sources^3,4^, because BDNF can cross the blood brain barrier in both directions^30^. Many investigations have been conducted on the value of blood BDNF as a biomarker of dementia, depression, and stroke^5–8^. Consistent with previous studies, we confirmed that plasma BDNF was associated with the presence of dementia and depression in this nationwide multicenter study.

BDNF and TrkB signaling in hypothalamus has a role for feeding behavior and energy homeostasis^31,32^. BDNF heterozygous mice exhibited abnormalities in eating behavior or locomotor activity and developed hyperplasia and obesity in early adulthood^33^. Additionally, BDNF administration had beneficial effects on glucose metabolism and insulin sensitivity in *db/db* mice and obese diabetic mice, but these effects of BDNF were not solely dependent on food intake^34,35^. In humans, individuals with type 2 diabetes showed low circulating levels of BDNF, and low plasma BDNF was associated with the severity of insulin resistance^9^. In their study, cerebral output of BDNF was abrogated during hyperglycemic clamp conditions, leading to decreased plasma BDNF levels. Another cohort study also showed that low levels of BDNF is an independent risk factor for diabetes and obesity^25,36^. On the other hand, diabetic patients older than 65 years were more likely to have higher prevalence of frailty than nondiabetic individuals^37,38^. In the present study, the association between BDNF and frailty persisted even after excluding individuals with diabetes.

Furthermore, BDNF may be a key mediator linking vascular and metabolic benefits of exercise with protection from neurodegenerative diseases^11^. The sources and mechanisms underlying exercise-induced increases in circulation levels of BDNF has not known well^14^. BDNF is a contraction-induced muscle cell-derived protein that can increase lipid oxidation in skeletal muscle though AMPK activation^15^. However, muscle-derived BDNF during physical exercise acts on the muscle in an autocrine and paracrine manner and does not release into circulation^15^. Platelets are known to take up BDNF from other sources via blood circulation, store it, and release it in response to stimuli such as traumatic muscle injury^4^. In addition, exercise is capable of increasing BDNF in various brain regions as well as circulating BDNF levels^18^, partly by induction of FNDC5 and its cleaved and secreted form irisin^16^. Therefore, it has been proposed that circulating BDNF levels reflect BDNF secreted by the brain in response to exercise^39^. Recently, it has been reported that lower blood BDNF levels are associated with skeletal muscle strength and physical performance, but not with muscle mass, in patients with heart failure^19^ and patients with hemodialysis^20^. Although loss of muscle mass and function in elderly can precede frailty, few studies were conducted on the association between plasma BDNF concentrations and frailty. Plasma BDNF concentrations were lower with frailty determined by Fried Frailty Phenotype and that plasma BNDF levels were normalized by exercise in Brazilian elderly women^18^. In addition, plasma BDNF levels were associated with frailty defined according to a Japanese version of CHS criteria in Japanese hemodialysis patients^20^. However, these studies were based on a small number of subjects and specific groups and did not evaluate the influence of cardiometabolic variables as well as the impact of associated chronic disorders, such as dementia, depression, stroke, diabetes, CVD, and osteoporosis. In the present study, we observed that plasm BDNF levels were significantly lower in participants with frailty. Furthermore, the presence of frailty was significantly associated with plasma BDNF levels even after extensive adjustment for confounding factors, including age and neurodegenerative diseases. Since the sensitivity of plasma BDNF in predicting frailty was low in the ROC analysis, it is necessary to use BDNF together with several other risk factors of frailty to increase sensitivity and specificity to increase the usefulness of BDNF as a screening test for frailty. Circulating levels of BDNF has been shown to decline with increasing age^24^. In sensitivity analyses after excluding dementia, depression, and insulin resistance-related metabolic diseases, which is closely related to BDNF, the association was maintained.

Our study has some limitations. Due to the intrinsic limitations of the cross-sectional study design, we could not determine the existence of a causal relationship between plasma BDNF and frailty. Decline in BDNF levels is accompanied by loss of muscle strength and physical performance caused by a decrease in physical activity and ageing, and as a result, it is thought to contribute to the development of frailty. Given the association between oxidative stress and frailty and the importance of oxidative stress in BDNF dysfunction^28,40^, interplay between BDNF and oxidative stress may contribute to the pathogenesis of frailty. Further studies are needed on the underlying mechanisms by which BDNF and TkrB signaling play a role in the development of frailty. Another limitation of the present study is that our study was limited to Korean men and women. Thus, it is difficult to apply our study results to different ethnic groups. Nevertheless, this study benefitted from using a nationally representative multicenter sample of community-dwelling elderly Korean adults from KFACS cohort database. Furthermore, multivariate analyses after adjusting for various confounding factors and extensive sensitivity analyses demonstrated consistent results, which may support the robustness of the present findings.

## Conclusions

We observed an association between plasma BDNF levels and frailty among community-dwelling older adults in Korea. Plasma BDNF levels were significantly lower in participants with frailty than in those without frailty. We also found a significant association between plasma BNDF levels and frailty, even after excluding participants with BDNF-related metabolic and neuropsychiatric disorders, such as dementia, depression, diabetes, CVD, stroke, and osteoporosis. Therefore, the results of our study may suggest the possible role of BDNF as a novel biomarker of frailty.

## Methods

### Data source and study population

This study was conducted using data from the Korean Frailty and Aging Cohort Study (KFACS), in which community-dwelling adults aged 70–84 years were recruited from 10 different medical centers nationwide using quota sampling methods stratified by age and sex^41^. Among 1,455 participants recruited to the KFACS in 2017, a total 717 individuals were included according to the following exclusion criteria: alcohol consumption > 14 drinks per week for men and > 7 drinks per week for women (n = 463), chronic liver diseases, including viral hepatitis and aspartate aminotransferase (AST) and alanine aminotransferase (ALT) levels greater than two-fold the upper limit of the normal levels (n = 21), blood creatinine level > 1.5 mg/dL (n = 25), any inflammatory disease with white blood cell count > 10,000/μL (n = 16), any kind of cancer (n = 19), and body composition data using bioelectrical impedance analysis (BIA) instead of dual-energy x-ray absorptiometry (DXA) measurements (n = 194). Of the remaining 717 participants, 317 individuals were randomly selected for the analysis of the plasma BDNF level. The final analysis included 302 participants, after 15 participants with missing data from an on-site interview about medical history and lifestyle factors and laboratory measurement.

### Assessment of frailty

Frailty was defined using the Fried Frailty Phenotype, also called Cardiovascular Health Study (CHS) Frailty Phenotype^21^ with modified cut-offs^41^, consisting of five components of frailty: unintentional weight loss (4.5 kg in past year), self-reported exhaustion, weakness (grip strength), slow walking speed, and low physical activity. Detailed information on above five components was described previously^41^. Grip strength was measured two times for each hand using a digital hand grip dynameter (Takei TKK 5401; Takei Scientific Instruments Co., Tokyo, Japan), and the highest value was used for the analysis. Gait speed was walking speed over 4 m using an automatic timer (Gaitspeedometer; Dynamicphysiology, Daejeon, Korea), with acceleration and deceleration phases of 1.5 m. The level of physical activity (kcal/week) was determined using the International Physical Activity Questionnaire (IPAQ) and metabolic equivalent scores were derived from vigorous, moderate, and mild activities in the questionnaire. Total CHS frailty scores were calculated by assigning a value of 1 to positive responses on each of the above five components (range: 0–5). Participants were considered as frail if the total score was 3–5 and non-frail if the total score was 0–2.

### Laboratory measurements

Blood samples were obtained at approximately 8 am after an 8-h fast in each center and transported to the core laboratory (Seegene Inc., Seoul, Korea). After centrifugation, the plasma samples were stored in a deep freezer until analysis. Blood chemistry tests including fasting plasma glucose (FPG), total cholesterol (TC), creatinine, hemoglobin, and high-sensitivity C-reactive protein (hs-CRP) were conducted using a Cobas 8000 C702 analyzer (Roche Diagnostics, Mannheim, Germany) except for plasma insulin. Plasma insulin was analyzed using a Cobas 8000 e602 analyzer (Roche Diagnostics) and HbA1c levels were measured using a Tosoh HLC-723 G8 analyzer (Tosoh Corporation, Tokyo, Japan). Homeostasis model assessment-insulin resistance (HOMA-IR) was calculated using the following formula: fasting plasma glucose (mmol/L) times fasting serum insulin (mU/L) divided by 405^42^. Plasma BDNF levels were measured using a quantitative sandwich enzyme-linked immunosorbent assay kit (catalog no. BEK-2211; Biosensis, Thebarton, Australia).

### Other variables and definitions

During their visit, the participants’ body weight, height, and systolic and diastolic blood pressure (SBP and DBP, respectively) were measured. Body mass index (BMI) was calculated as body weight (kg) divided by height squared in meters (m^2^). Information on age, sex, smoking status, alcohol consumption, physical activity, income, and medical history (including previous diagnosis of diabetes, hypertension, dyslipidemia, stroke, angina, myocardial infarction and osteoporosis) of the participants was assessed by on-site interviews. Regular drinking was defined as alcohol intake ≥ 2 times/week. Income levels were dichotomized at the lower 20% level. Obesity was defined as BMI ≥ 25 kg/m^2^. Chronic kidney disease was defined as an estimated glomerular filtration rate < 60 mL/min/1.73 m^2^ calculated by the Chronic Kidney Disease Epidemiology Collaboration Eq. ^43^. Diabetes was defined as FPG ≥ 126 mg/dL, HbA1c ≥ 6.5%, or previous diagnosis of diabetes. CVD was defined as a previous diagnosis of both angina and myocardial infarction. Cognitive function was evaluated using the Mini-Mental State Examination (MMSE), and dementia was defined as a MMSE score < 24^44^. Depression was determined using the Korean version of the Short Form Geriatric Depression Scale with a score of ≥ 6^45^.

### Statistical analysis

Data are presented as the means ± standard deviations or median (interquartile range) for continuous variables, and as counts and percentages (%) for categorical variables. Comparisons of baseline characteristics between participants with and without physical frailty were performed using independent two-sample t-test or Mann-Whiney U-test for continuous variables and the χ^2^ test for categorical variables. Because of the highly skewed distribution in levels of plasma BDNF, the natural log transformation of the data was used for statistical analysis. Univariate and multivariate logistic regression analyses for the presence of physical frailty as the dependent variable and plasma BNDF value, age, sex, BMI, SBP, current smoking, regular drinking, low income, and laboratory data including TC, FPG, HOMA-IR, hs-CRP, hemoglobin, creatinine, and dementia and depression as the independent variables. In a multivariate logistic regression model, all factors that were found to be significant in univariate analyses were used. We also conducted sensitivity analyses to exclude the effects of BDNF-related comorbidities such as dementia, depression, stroke, diabetes, CVD, and osteoporosis. Receiver operating characteristic (ROC) analysis was used to verify the predictive validity of plasma BDNF for estimating frailty. A P–value < 0.05 was considered statistically significant throughout the analysis. All statistical analyses were conducted by an experienced professional statistician using SAS version 9.4 (SAS Institute Inc., Cary, NC, USA).

### Ethics

The study was performed according to the 1964 Declaration of Helsinki. The study protocol for the KFACS was approved by the institutional review board of the Korea University Guro Hospital (Approval no. 2020GR0139). Written informed consents were obtained from all participants.

## Supplementary Information

Supplementary Information.

## Data Availability

The data used to support the findings of this study are available from the corresponding author on reasonable request.

## Abbreviations

BMI: Body mass index
BDNF: Brain-derived neurotrophic factor
CHS: Cardiovascular Health Study
CI: Confidence interval [CI]
CVD: Cardiovascular disease
HOMA-IR: Homeostasis model assessment-insulin resistance
hs-CRP: High-sensitivity C-reactive protein
KFACS: Korean Frailty and Aging Cohort Study
OR: Odds ratio
