# Supplementary material for: Association of plasma brain-derived neurotrophic factor levels and frailty in community-dwelling older adults
Source: Sci Rep. 2022 Nov 3;12:18605. doi: 10.1038/s41598-022-19706-3 (PMC9633836; doi:10.1038/s41598-022-19706-3)
Supplement: Supplementary file 1 — Supplementary Information. [file 41598_2022_19706_MOESM1_ESM.docx]

**Association of plasma brain-derived neurotrophic factor levels and frailty in community-dwelling older adults: The Korean Frailty and Aging Cohort Study**

Eun Roh^1^, Soon Young Hwang^2^, Eyun Song, MD^3^, Min Jeong Park^3^, Hye Jin Yoo, MD^3^, Sei Hyun Baik^3^, Miji Kim^4^, Chang Won Won^5*^, Kyung Mook Choi^3*^

^*^CWW and KMC contributed equally to this work.

^1^Division of Endocrinology and Metabolism, Department of Internal Medicine, Hallym University Sacred Heart Hospital, Anyang, Republic of Korea; ^2^Department of Biostatistics, Korea University College of Medicine, Seoul, Republic of Korea; ^3^Division of Endocrinology and Metabolism, Department of Internal Medicine, Korea University College of Medicine, Seoul, Republic of Korea; ^4^East-West Medical Research Institute, Kyung Hee University, Seoul, Republic of Korea: ^5^Department of Family Medicine, College of Medicine, Kyung Hee University, Seoul, Republic of Korea

**Supplementary Table 1.** Association of brain-derived neurotrophic factor or metabolic parameters with the presence of frailty after excluding subjects with dementia, depression, stroke, diabetes, cardiovascular disease, or osteoporosis.

|  | Univariate | |  | Multivariate | |
| --- | --- | --- | --- | --- | --- |
|  | OR (95% CI) | *P*-value |  | OR (95% CI) | *P*-value |
| Excluding subjects with dementia (n = 234) | | | | | |
| Age | 1.137 (0.997, 1.297) | 0.056 |  |  |  |
| Sex | 0.722 (0.275, 1.894) | 0.508 |  |  |  |
| BMI | 1.066 (0.919, 1.236) | 0.400 |  |  |  |
| SBP | 0.979 (0.949,1.011) | 0.198 |  |  |  |
| Current smoking | <.001  (<.001, >999.999) | 0.973 |  |  |  |
| Regular drinking | 0.484 (0.108, 2.179) | 0.345 |  |  |  |
| Low income | 1.491 (0.555, 4.005) | 0.428 |  |  |  |
| TC | 1.002 (0.989, 1.016) | 0.757 |  |  |  |
| FPG | 1.004 (0.989, 1.02) | 0.568 |  |  |  |
| HOMA-IR | 0.993 (0.831, 1.186) | 0.936 |  |  |  |
| hs-CRP | 0.847 (0.52, 1.38) | 0.504 |  |  |  |
| Hemoglobin | 0.709 (0.48, 1.047) | 0.084 |  |  |  |
| Creatinine | 1.443 (0.165, 12.649) | 0.741 |  |  |  |
| BDNF | 0.525 (0.277, 0.994) | 0.048 |  | 0.51 (0.257, 1.013) | 0.055 |
| Depression | 5.568 (1.973, 15.714) | 0.001 |  | 5.525 (1.932, 15.802) | 0.001 |
| Excluding subjects with depression (n = 235) | | | | | |
| Age | 1.159 (1.012, 1.326) | 0.033 |  | 1.115 (0.965, 1.289) | 0.038 |
| Sex | 1.236 (0.455, 3.357) | 0.678 |  |  |  |
| BMI | 0.908 (0.782, 1.055) | 0.209 |  |  |  |
| SBP | 0.992 (0.959, 1.026) | 0.634 |  |  |  |
| Current smoking | 1.607 (0.341, 7.587) | 0.549 |  |  |  |
| Regular drinking | 0.225 (0.029, 1.741) | 0.153 |  |  |  |
| Low income | 1.141 (0.407, 3.202) | 0.802 |  |  |  |
| TC | 0.994 (0.98, 1.008) | 0.404 |  |  |  |
| FPG | 1.009 (0.996, 1.022) | 0.184 |  |  |  |
| HOMA-IR | 0.968 (0.749, 1.25) | 0.801 |  |  |  |
| hs-CRP | 0.997 (0.796, 1.249) | 0.978 |  |  |  |
| Hemoglobin | 0.739 (0.503, 1.084) | 0.121 |  |  |  |
| Creatinine | 4.752 (0.577, 39.148) | 0.148 |  |  |  |
| BDNF | 0.515 (0.277, 0.959) | 0.037 |  | 0.511 (0.273, 0.958) | 0.036 |
| Dementia | 4.582 (1.56, 13.461) | 0.006 |  | 4.077 (1.296, 12.823) | 0.016 |
| Excluding subjects with stroke (n = 254) | | | | | |
| Age | 1.194 (1.07, 1.332) | 0.002 |  | 1.164 (1.029, 1.316) | 0.015 |
| Sex | 0.894 (0.409, 1.956) | 0.780 |  |  |  |
| BMI | 0.998 (0.888, 1.121) | 0.967 |  |  |  |
| SBP | 0.985 (0.959,1.011) | 0.259 |  |  |  |
| Current smoking | 1.573 (0.433, 5.715) | 0.491 |  |  |  |
| Regular drinking | 0.466 (0.135, 1.604) | 0.226 |  |  |  |
| Low income | 1.953 (0.891, 4.283) | 0.095 |  |  |  |
| TC | 1.002 (0.992, 1.013) | 0.660 |  |  |  |
| FPG | 1.008 (0.998, 1.019) | 0.126 |  |  |  |
| HOMA-IR | 1.008 (0.893, 1.139) | 0.893 |  |  |  |
| hs-CRP | 1.059 (0.929, 1.208) | 0.390 |  |  |  |
| Hemoglobin | 0.681 (0.504, 0.922) | 0.013 |  | 0.776 (0.559, 1.076) | 0.128 |
| Creatinine | 4.836 (0.906, 25.799) | 0.065 |  |  |  |
| BDNF | 0.508 (0.295, 0.874) | 0.015 |  | 0.537 (0.291, 0.991) | 0.047 |
| Dementia | 3.365 (1.439, 7.87) | 0.005 |  | 1.442 (0.512, 4.061) | 0.488 |
| Depression | 5.608 (2.455, 12.807) | <0.001 |  | 4.165 (1.675, 10.358) | 0.002 |
| Excluding subjects with diabetes (n = 178) | | | | | |
| Age | 1.139 (0.993, 1.307) | 0.064 |  |  |  |
| Sex | 1.094 (0.393, 3.044) | 0.863 |  |  |  |
| BMI | 1.051 (0.904, 1.221) | 0.520 |  |  |  |
| SBP | 0.976 (0.944,1.009) | 0.151 |  |  |  |
| Current smoking | 1.673 (0.345, 8.116) | 0.523 |  |  |  |
| Regular drinking | 0.91 (0.246, 3.365) | 0.888 |  |  |  |
| Low income | 3.733 (1.243, 11.215) | 0.019 |  | 3.244 (0.968, 10.874) | 0.057 |
| TC | 1.002 (0.987, 1.017) | 0.835 |  |  |  |
| FPG | 1.018 (0.962, 1.076) | 0.542 |  |  |  |
| HOMA-IR | 1.25 (0.773, 2.02) | 0.363 |  |  |  |
| hs-CRP | 1.077 (0.916, 1.266) | 0.370 |  |  |  |
| Hemoglobin | 1.129 (0.743, 1.714) | 0.570 |  |  |  |
| Creatinine | 3.171 (0.279, 36.03) | 0.352 |  |  |  |
| BDNF | 0.384 (0.172, 0.853) | 0.019 |  | 0.345 (0.14, 0.849) | 0.021 |
| Dementia | 3.063 (0.976, 9.619) | 0.055 |  |  |  |
| Depression | 8.368 (2.815, 24.874) | <0.001 |  | 6.159 (1.922, 19.736) | 0.002 |
| Excluding subjects with CVD (n = 252) | | | | | |
| Age | 1.206(1.08,1.347) | 0.001 |  | 1.149(1.017,1.299) | 0.026 |
| Sex | 0.908(0.419,1.966) | 0.807 |  |  |  |
| BMI | 1.009(0.901,1.13) | 0.877 |  |  |  |
| SBP | 0.989(0.964,1.015) | 0.406 |  |  |  |
| Current smoking | 1.415(0.392,5.106) | 0.596 |  |  |  |
| Regular drinking | 0.49(0.143,1.687) | 0.258 |  |  |  |
| Low income | 1.863(0.861,4.033) | 0.114 |  |  |  |
| TC | 1(0.989,1.011) | 0.987 |  |  |  |
| FPG | 1.009(0.998,1.02) | 0.118 |  |  |  |
| HOMA-IR | 1.012(0.902,1.135) | 0.837 |  |  |  |
| hs-CRP | 1.061(0.934,1.206) | 0.361 |  |  |  |
| Hemoglobin | 0.798(0.593,1.073) | 0.135 |  |  |  |
| Creatinine | 3.944(0.707,22.008) | 0.118 |  |  |  |
| BDNF | 0.501(0.289,0.867) | 0.014 |  | 0.467(0.255,0.857) | 0.014 |
| Dementia | 4.526(1.988,10.303) | <0.001 |  | 2.713(1.036,7.106) | 0.042 |
| Depression | 5.038(2.231,11.372) | <0.001 |  | 3.616(1.476,8.86) | 0.005 |
| Excluding subjects with osteoporosis (n = 259) | | | | | |
| Age | 1.219 (1.077,1.379) | 0.002 |  | 1.12 (0.971,1.292) | 0.120 |
| Sex | 1.062 (0.448,2.517) | 0.892 |  |  |  |
| BMI | 0.943 (0.828,1.074) | 0.376 |  |  |  |
| SBP | 1.002 (0.974,1.031) | 0.874 |  |  |  |
| Current smoking | 1.635 (0.447,5.981) | 0.458 |  |  |  |
| Regular drinking | 0.537 (0.153,1.876) | 0.330 |  |  |  |
| Low income | 1.437 (0.604,3.417) | 0.412 |  |  |  |
| TC | 1 (0.988,1.013) | 0.937 |  |  |  |
| FPG | 1.009 (0.998,1.02) | 0.122 |  |  |  |
| HOMA-IR | 1.016 (0.902,1.144) | 0.797 |  |  |  |
| hs-CRP | 1.053 (0.914,1.212) | 0.474 |  |  |  |
| Hemoglobin | 0.776 (0.562,1.07) | 0.122 |  |  |  |
| Creatinine | 7.334 (1.189,45.244) | 0.032 |  | 8.929 (1.164,68.479) | 0.035 |
| BDNF | 0.434 (0.237,0.795) | 0.007 |  | 0.445 (0.232,0.855) | 0.015 |
| Dementia | 4.779 (1.938,11.785) | 0.001 |  | 3.132 (1.053,9.312) | 0.040 |
| Depression | 4.293 (1.713,10.756) | 0.002 |  | 3.511 (1.191,10.35) | 0.023 |

Univariate and multivariate logistic regression analyses for the presence of physical frailty as the dependent variable and plasma BNDF value, age, sex, BMI, SBP, lifestyle factors, laboratory data, and dementia and depression as the independent variables. In a multivariate logistic regression model, all factors that were found to be significant in univariate analyses were used. BDNF, brain-derived neurotrophic factor; BMI, body mass index; CI, confidence interval; CVD, cardiovascular disease; FPG, fasting plasma glucose; HOMA-IR, homeostasis model assessment-insulin resistance; hs-CRP, high-sensitivity C-reactive protein; OR, odds ratio; SBP, systolic blood pressure; TC, total cholesterol.
